# United States house dust Pb concentrations are influenced by soil, paint, and house age: insights from a national survey

**DOI:** 10.1038/s41370-024-00655-0

**Published:** 2024-03-28

**Authors:** Tyler D. Sowers, Clay M. Nelson, Matthew D. Blackmon, Kevin Li, Marissa L. Jerden, Alicia M. Kirby, Kasey Kovalcik, David Cox, Gary Dewalt, Warren Friedman, Eugene A. Pinzer, Peter J. Ashley, Karen D. Bradham

**Affiliations:** 1 Center of Environmental Measurement and Modeling, Office of Research and Development, US Environmental Protection Agency, Research Triangle Park, NC 27711 USA; 2 BioGeoChem Scientific, Austin, TX 78748 USA; 3 Independent Researcher, Lansing, MI 48915 USA; 4 Jacobs Technology, Inc., 109 T.W. Alexander Drive, Research Triangle Park, NC 27711 USA; 5 Oak Ridge Associated Universities, Oak Ridge, TN 37830 USA; 6 QuanTech, 6110 Executive Blvd Suite 206, Rockville, MD 20852 USA; 7 Office of Lead Hazard Control and Healthy Homes, Department of Housing and Urban Development, Washington, DC 20410 USA

**Keywords:** Lead, Lead dust, House dust, Soil, Paint, Lead poisoning

## Abstract

**Background:**

Lead (Pb) in house dust contributes significantly to blood lead levels (BLLs) in children which may result in dire health consequences. Assessment of house dust Pb in the United States, relationships with Pb in soil and paint, and residential factors influencing Pb concentrations are essential to probing drivers of house dust Pb exposure.

**Objective:**

Pb concentrations in vacuum-collected house dust are characterized across 346 homes participating in the American Health Homes Survey II (AHHS II), a US survey (2018–2019) evaluating residential Pb hazards. Connections between house dust Pb and soil Pb, paint Pb, and other residential factors are evaluated, and dust Pb concentration data are compared to paired loading data to understand Pb hazard standard implications.

**Results:**

Mean and median vacuum dust Pb concentrations were 124 µg Pb g^−1^ and 34 µg Pb g^−1^, respectively. Vacuum-collected dust concentrations and dust wipe Pb loading rates were significantly correlated within homes (*α* < 0.001; *r* ≥ 0.4). At least one wipe sample exceeded current house dust Pb loading hazard standards (10 µg ft^−2^ or 100 µg Pb ft^−2^ for floors and windowsills, respectively) in 75 of 346 homes (22%). House dust Pb concentrations were correlated with soil Pb (*r* = 0.64) and Pb paint (*r* = 0.57). Soil Pb and paint Pb were also correlated (*r* = 0.6).

**Impact:**

The AHHS II provides a window into the current state of Pb in and around residences. We evaluated the relationship between house dust Pb concentrations and two common residential Pb sources: soil and Pb-based paint. Here, we identify relationships between Pb concentrations from vacuum-collected dust and paired Pb wipe loading data, enabling dust Pb concentrations to be evaluated in the context of hazard standards. This relationship, along with direct ties to Pb in soil and interior/exterior paint, provides a comprehensive assessment of dust Pb for US homes, crucial for formulating effective strategies to mitigate Pb exposure risks in households.

## Introduction

House dust, soil, and drinking water are important sources of residential lead (Pb) exposure, a metal contaminant that can lead to dire health consequences, especially in children younger than 6 years old [1–4]. To better understand Pb levels in United States (US) homes, the American Healthy Homes Survey (AHHS) II was conducted (2018–2019) via a collaboration between the United States Environmental Protection Agency (EPA) and the Department of Housing and Urban Development (HUD) [3]. While its primary focus was to monitor changes in the prevalence of Pb-based paint in homes, AHHS II also included sampling of house dust, soil and drinking water from residences where children reside. Additionally, residential data was collected, enabling assessment of potential connections between contaminants and residential characteristics (e.g., house age, location of residence, etc.).

Pb-exposure in children can impede brain development, resulting in permanently diminished IQ [5, 6]. This repercussion is important as residential sources of Pb contamination continue to drive elevated blood lead levels (BLLs) globally [5–7], and may raise environmental justice concerns as Pb-contamination may disproportionally impact the intellectual potential of disadvantaged communities [7–11]. Therefore, significant health and socioeconomic consequences may be driven by early life Pb exposure, making elucidation of Pb prominence and correlations to residential sources a vital research need.

Exposure to Pb via house dust in US homes and its continued impact on BLLs in children remains of paramount concern. Variation in house dust Pb is nuanced given matrix complexity and characterization difficulty [12–14]. House dust is most commonly a mixture of biological organics, soils, household products, and/or building materials that may contain variable contaminant phases [15–18]. Associations between Pb in dust with Pb in soil, paint, and other residential factors need further investigation in the US, especially when considering processes driving elevated dust Pb concentrations in homes. Exterior soil and indoor residential materials (e.g., Pb-based paint) have been examined to determine sources of house dust Pb [17–20]. Urban soils are currently thought to be major mediators of house dust Pb due to proximity to industry and legacy use of Pb-containing products. While use of Pb-based paint in the US has decreased over the last several decades, Pb-based paint has still been found to be an important mediator of house dust Pb [20–23]. Lead-based paint hazards are defined in federal law as including deteriorated lead paint, lead-contaminated bare soil, and lead contaminated settled house dust; therefore, there are multiple pathways of Pb exposure concern. Elucidation of residential relationships with Pb concentrations in house dust is critical to safeguarding children due to the strong connection between house dust Pb and elevated BLLs (>5 µg dl^−1^) [17, 24, 25]. Every action to prevent children from coming into contact with Pb is important and can improve their health and lives, as no safe blood lead level in children has been identified [25].

House dust collection for Pb analyses typically consists of either vacuum-collection or dust wipe analysis. Vacuum-collected house dust is expressed on a Pb concentration basis and notated as Pb mass per composited mass of house dust (i.e., µg Pb g^−1^ dust), whereas dust wipe data is expressed as Pb loading data represented as mass of Pb relative to surface area sampled (typically, a floor or windowsill) (i.e., µg ft^−2^ in the US) [26, 27]. House dust Pb hazard standards in the US are in units of Pb loading due to strong correlation with BLLs in children [24]. In January 2020, EPA announced clearance levels of 10 µg Pb ft^−2^ for floor dust and 100 µg Pb ft^−2^ for windowsill dust, a recent change from 40 µg Pb ft^−2^ and 250 µg Pb ft^−2^ [28]. While dust Pb loading data is used as the basis for hazard standards, vacuum-collected data may be more suitable for probing connections to other media. This is due to more available material for characterization, including speciation and bioaccessibility/bioavailability analyses [14, 21, 29–31]. Importantly, there is uncertainty regarding how house dust Pb concentration relates to human health and an absence of house dust Pb hazard standards based on concentration. Rarely are both dust Pb concentrations and loading assessed in the same investigation and recent investigations of Pb in house dust similar in scope to AHHS II are limited. A series of investigations conducted by Health Canada provide a recent assessment of house dust Pb via determination of both dust Pb loadings and dust Pb concentrations [21, 29–31]. Health Canada investigations address advantages to both, allowing for determination of Pb mineralogical phases and linkage to current house dust Pb health standards. House age and urbanization were found to be drivers of Pb in these investigations [21].

Significant knowledge gaps remain regarding house dust Pb in the US, which are essential for a comprehensive understanding of residential Pb exposure. In this study, we present findings of Pb in vacuum-collected house dust sampled from homes participating in AHHS II and draw associations to soil Pb and paint Pb data previously reported by HUD [28]. Specifically, this study aims to (1) present Pb concentrations in vacuum-collected house dust for 346 US homes participating in the AHHS II, (2) compare dust Pb concentration data to house dust Pb loading data per residential surface area sampled to understand hazard standard implications, and (3) evaluate connections between house dust Pb and soil Pb, paint Pb, and other residential factors.

## Methods

### Sample collection

As part of AHHS II, dust samples to be analyzed for Pb concentration were collected from personal household vacuums. Additionally, house dust wipe data was collected by HUD for the same residences [28], allowing for data comparison. Samples collected from vacuum bags represent, among other factors, an array of vacuum type/components used (e.g., bagless vacuums, bagged vacuums, various vacuum filters, etc.), areas of the home vacuumed, vacuuming frequency, and time since last vacuuming. A field collection team was responsible for collecting samples in sealable, uniform, plastic bags. Vacuum bags were voluntarily provided from 554 of the 678 homes that participated in AHHS II. There are numerous methodologies for collecting house dust using vacuum sampling methods; therefore, the term “vacuum-collected house dust” hereafter refers to the AHHS II house dust collection methods reported in this study unless otherwise noted. The AHHS II included a questionnaire asking participants about their residential environment along with information collected by the field study team regarding the residence that was sampled. Questionnaire data were collected from residents; however, not all respondents provided answers to the questionnaire, not all homeowners provided vacuum dust contents, and some vacuum dust contents contained insufficient mass for analysis, which created a smaller vacuum dust data set to evaluate than the dust wipe data set collected and analyzed by HUD. Upon review of the data available, we determined the AHHS II vacuum data set lacked the statistical power needed to explore statistical relationships between Pb in dust and questionnaire data. Analysis of region and house age did not require responses from the homeowner/representative. For more detail about the questionnaire and data collected during the AHHS II field collection, refer to HUD’s AHHS II final report [28].

### Sample analysis

Upon completion of field collection, sealed samples were shipped by the field sampling team under chain-of-custody to the USEPA’s Office of Research and Development (ORD) in Durham, NC for analysis. A total of 554 dust samples were received. Collection bag IDs were inventoried and matched to chain-of-custody reports included with each shipment. Samples with low sample mass (<5 g) and/or compositions consisting primarily of recognizable materials (e.g., hair, insects, nail clippings, etc.) were excluded from further analysis. The remaining 346 samples were sieved through stacked 2 mm (#10) and 150 µm (#100) mesh size sieves. Sieved samples were stored in 250 ml HDPE containers until further analysis.

The 346 sieved dust samples were digested by either microwave or hotblock digestion and analyzed for total Pb concentration using <150 µm house dust. This particle size is the fraction best associated with hand-to-mouth Pb exposure [32]. Sample mass for all digestions was 0.5 g (±10%) with the exception of mass-limited samples, in which case 0.25 g (±10%) samples were used. In total, 262 samples were analyzed by hotblock digestion either by EPA or under EPA contract (RTI International). Hotblock digestion was performed using in-house distilled (subPUR) trace metal grade concentrated nitric acid. Samples were digested at an initial holding time of 30-min at 60 °C then ramped to 95 °C for a 1-h hold-time. A subsequent hydrogen peroxide polish was performed for 1 h at 95 °C. Quality control (QC) samples associated with each hotblock digestion batch included a blank, blank spike, NIST SRM (SRM 2710a or 2583), matrix spike, and sample duplicates. Eighty-four dust samples were digested at EPA by microwave digestion according to EPA Method 3051 (MARS; CEM; Matthews, NC) [33]. Microwave digestion was not used for the entire study due to considerable time constraints associated with microwave digestion versus hotblock digestion. Similar QCs were used for all digestions. Results of QC sample recoveries used across digestion methods and labs indicate that results were not significantly influenced by digestion method or analytical lab (SI Table S1). Analysis of all digested samples was performed via inductively coupled plasma-mass spectrometer (iCAP RQ; Thermo Scientific) in accordance with EPA Method 6020b [34].

### Wipe, paint and soil data

Vacuum-collected house dust Pb concentrations were compared to other data collected as part of the AHHS II and analyzed by HUD, including dust wipe (i.e., Pb loading data), soil Pb concentration, and paint Pb X-ray fluorescence (XRF) datasets. Dust wipes and soils were digested via hotblock and analyzed via ICP-MS according to EPA Methods 3050b and 6020b [34, 35]. A summary of the XRF and dust wipe collection methods used by HUD are included in the SI. Further details regarding methodologies for analyses and associated data for dust wipes, soil Pb, and paint Pb XRF are reported in a recent HUD report [28].

### Statistical analyses

Statistical analyses were performed using R version 4.0.1 (R Development Core Team 2020). Log-linear regression was used to evaluate the association between house age and dust-Pb concentrations (log-transformed) across study homes. Associations between dust-Pb concentrations and US census region were fit in log-linear multiple regressions controlling for house age. The significance of the geographical region of the home, controlling for house age, was tested using a likelihood ratio test comparing the more complex model against the simple log-linear regression with home age only, using the R package “lmtest” [36]. The pairwise differences in the model-predicted marginal means of dust Pb levels in each census region (holding the effect of house age constant) were also tested using the Tukey post hoc test, with the R package “emmeans” [37].

To relate Pb dust concentrations (µg g^−1^) collected from vacuum bags to health-based hazard standards, concentration data are compared to paired wipe data collected by HUD. To inform sources of Pb in dust, dust Pb levels are related to paired Pb paint and soil Pb data collected from each home [28]. Associations between dust Pb concentration data (vacuum-collected) and paired Pb loading data (dust wipe), soil Pb, and Pb paint were evaluated using Pearson’s coefficient of correlation (*r*). In cases where multiple samples of Pb wipe loading (per individual house area or surface), soil Pb, and paint Pb were collected, arithmetic means of these samples were used to correlate with vacuum Pb dust concentration data. Arithmetic mean was used for all mean values reported. To relate observed dust Pb concentrations to Pb loading rate-based US hazard standards, logistic regression models were developed to relate measured vacuum dust Pb concentration in each home to the probability of an exceedance of US hazard standards assessed using Pb wipe loading data collected from the same home. Three regressions were performed, each estimating probability of exceedance of at least one wipe sample from within the subset of (1) floor wipes (>10 µg Pb ft^−2^), (2) windowsill wipes (>100 µg Pb ft^−2^), or (3) across all floor and windowsill wipes.

## Results

### Pb contamination of United States house dust

Of the 703 homes that participated in AHHS II, a vacuum bag sample of sufficient mass for analysis was provided from 346 homes. Mean and median Pb concentrations in sampled vacuum bags were 124 µg Pb g^−1^ and 34 µg Pb g^−1^, respectively (Table 1).

**Table 1:** Summary statistics for house dust, soil, and paint Pb.

Matrix and/or sampling location	*n*	Mean	Median	25% quantile	75% quantile	95% quantile
House dust Pb concentration: vacuum-collected (µg Pb g^−1^)						
Vacuum bags^a^	346 homes^b^	124	34	17	67	379
House dust Pb loading: dust wipes (µg Pb ft^−2^)						
All	337 homes	22.3	1	0.2	5	62.3
Floor only		5.2	0.4	0.1	1.6	12.8
Window only		51	1.8	0.4	8	132
Pb in paint (mg Pb cm^−2^)						
All	337 homes	0.39	0.12	0.09	0.23	3.18
Exterior paint		0.66	0.12	0.08	0.3	6.4
Interior paint		0.33	0.12	0.09	0.18	2.6
Pb in soil (µg Pb g^−1^)						
All	295 homes	135.3	25.1	14.3	67.7	978.5
Foundation drip line		175.4	28.6	15.7	84.9	1073
Major entryway		142.2	27.1	14.6	65.4	986.9
Mid-yard		80.4	21.7	13.7	57.6	599.7
Children’s play area		79	19.8	11.6	45.8	748

House dust Pb is presented as µg Pb g^−1^ dust for vacuum-collected house dust, whereas dust wipe data are presented as µg ft^−2^. Locations for dust wipe, paint, and soil Pb data are provided. Pb in paint was determined via handheld XRF.

^a^Household vacuum cleaner bags.

^b^Nine homes included in the dust Pb concentration dataset did not have a corresponding dust wipe collected.

### Residential indicators of house dust Pb

**Fig. 1:**
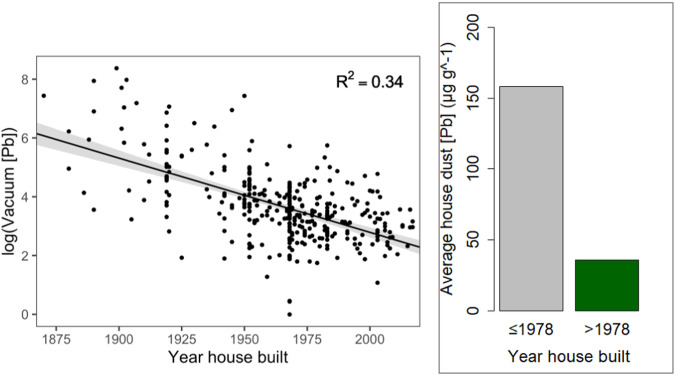
Linear regression showcasing log (house dust [Pb]) with year homes were built for AHHS II homes evaluated for vacuum-collected Pb concentration (log [vac Pb] = −0.02 × house age (year built) + 53.3; *r*^2^ = 0.34). Mean [Pb] is shown ≤1978 and >1978 to highlight decreases in house dust Pb concentration after the banning of Pb-based paint in the United States in 1978.

Pb dust concentrations observed in this study were significantly correlated with house age (*p* < 0.001, *r*^2^ = 0.34), with older homes associated with higher dust Pb concentrations (Fig. 1). The log-linear model estimated dust Pb concentration decreased by 2.5% (95% CI = 2.1–2.8%, *p* < 0.001) with every year a house was more recently built. The mean dust Pb concentration from homes built in or before 1978 was 158 µg Pb g^−1^, versus 36 µg Pb g^−1^ from homes built after 1978, the year after the U.S. Consumer Product Safety Commission (CPSC) reduced the allowable concentration of Pb in house paint to 0.06% by weight [38].

**Fig. 2:**
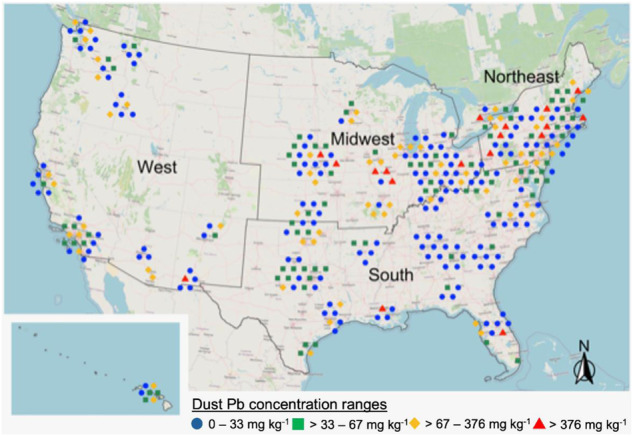
House dust Pb concentration results overlaid on a map of the United States. Map was constructed using the R packages “ggplot”, “sf”, and “ggspatial”. Scatter was applied to sample locations to avoid overlapping of symbols.

Dust Pb concentrations were also evaluated by US census region (Northeast, Midwest, South or West), controlling for house age, to explore if geographic factors beyond house age may impact observed dust Pb concentrations (Figs. 2 and S1). Mean and median dust Pb concentrations, respectively, for US census regions were 188 and 44 µg Pb g^−1^ for the Northeast, 189 and 40 µg Pb g^−1^ for the Midwest, 67 and 29 µg Pb g^−1^ for the South, and 60 and 29 µg Pb g^−1^ for the West. A multiple regression analysis, using house age and region as predictors of mean vacuum dust Pb concentrations, found there were significant differences (*p* < 0.05) in vacuum dust Pb by region beyond those that would be explained by house age alone (see SI). These may include factors such as traffic density, or average distance to industry [12, 39]; however, such additional factors were not evaluated as part of this study. Dust Pb concentrations were similarly evaluated based on whether sampled homes were within a Metropolitan Statistical Area (MSA), generally defined by the US Census Bureau as a geographical entity comprised of one or more counties with at least one urbanized area of 50,000 or more inhabitants [40]. However, results did not indicate MSA was a significant explanatory variable to predict dust Pb levels observed in this survey (SI Fig. S2).

### Connecting dust Pb concentration to dust Pb loading datasets

We evaluated the relationship between vacuum collected house dust data (µg Pb g^−1^) and dust wipe data (µg Pb ft^−2^) using Pearson’s coefficient of correlation (*r*) (SI Table S2). Across most locations, paired vacuum-collected and wipe data was significantly correlated (*α* < 0.001; *r* ≥ 0.4). Of any single collection area from a specific room, vacuum Pb concentration was most strongly correlated with entryway floor wipe samples (*r* = 0.44). Correlations with windowsills were weaker, especially in the kitchen (*r* = 0.26). Combining wipe data by surface (i.e., all floors or all windowsills) improved correlations with vacuum data (*r* = 0.48, 0.41 and 0.49 for the mean, median and 95th quantile for combined floor wipe values and 0.41, 0.40 and 0.41 for mean, median and 95th quantile for combined windowsill wipe values).

### Contextualizing house dust Pb concentrations via current hazard standards

**Fig. 3:**
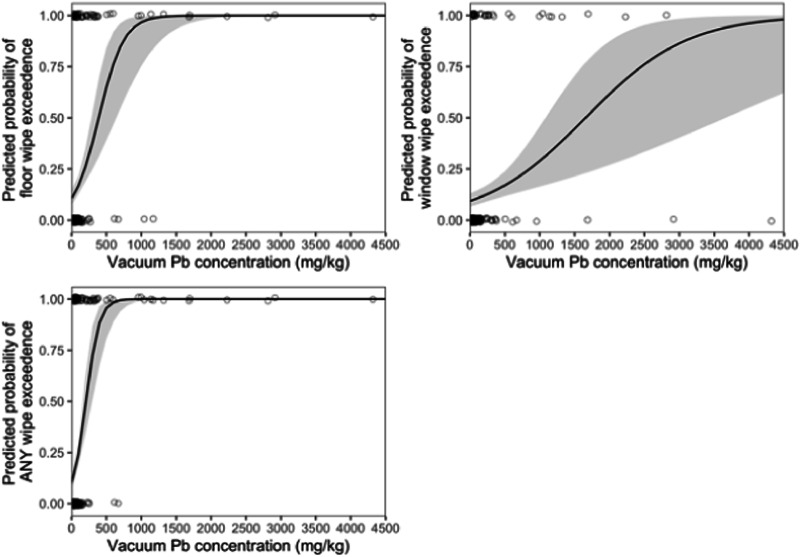
Predicted probability of dust Pb exceedence for a given house dust Pb concentration. Predicted probability of at least one floor wipe (top-left), window wipe (top-right), or any wipe (bottom) exceedence of the 10 µg ft^−2^ and 100 µg ft^−2^ US Pb dust hazard standard for floor and window wipes, respectively, as a function of dust Pb concentration collected from vacuum bags.

Logistic regression was used to estimate probabilities of observing at least one floor, window, or either wipe exceedance based on observed vacuum Pb concentration values (Fig. 3). In instances where only dust Pb concentration data is available, these regression models may be useful to statistically relate vacuum dust Pb concentrations in a home to probability of observing a dust Pb hazard standard exceedance based on Pb surface loading. Notably, homes with vacuum Pb concentrations of ~1000 µg Pb g^−1^ had a near 100% probability of having at least one floor wipe sample in exceedance of the 10 µg Pb ft^−2^ hazard standard, but only an ~25% probability of having at least one windowsill wipe sample that exceeded the 100 µg Pb ft^−2^ hazard standard. For all wipe data, regardless of location, house dust Pb concentration of ~400 µg Pb g^−1^ had a ~75% probability of an exceedance.

Additionally, homes were binned based on the fraction of wipe samples that exceeded current dust Pb loading hazard standards and related to average Pb concentration (Table 2). Seventy-five of 337 homes (22%) had at least one wipe sample that exceeded the US Pb dust hazard standard for floors (>10 µg Pb ft^−2^). Mean house dust Pb concentration across these 75 homes was 416 µg Pb g^−1^, compared to a mean Pb dust concentration of 44 µg Pb g^−1^ from the 262 (of 337) homes with no wipe exceedances. Twenty-four of 337 homes (7%) had both a floor wipe and window wipe exceedance. Mean dust Pb concentrations across these 24 homes was 575 µg Pb g^−1^. Thirty-seven of 337 (11%) and 14 of 337 (4%) homes had only a floor wipe or a window wipe exceedance (but not both), with mean dust Pb concentrations of 387 and 221 µg Pb g^−1^, respectively.

**Table 2:** Percent of homes exceeding current house dust Pb loading hazard standards of 10 µg Pb ft^−2^ and 100 µg Pb ft^−2^ for floors and windowsills, respectively.

Dust Pb datasets	*n*	Percent of homes (%)	Mean dust [Pb] (µg Pb g^−1^)
Floor or window	75	22	416
Floor only	37	11	387
Window only	14	4	221
Floor and window	24	7	575
No exceedance	262	78	44
All homes	337	100	127

Average house dust Pb concentration is reported for each category, providing insight into current dust concentrations in homes when either exceeding or meeting current hazard standards.

### Evaluating correlations of house dust Pb with soil and paint Pb

Residential soil collected from the surrounding property at multiple locations along with exterior and interior paint Pb were investigated as contributors to house dust Pb [28]. Mean and median soil Pb concentrations across all sampled homes were 135.3 and 25.1 µg Pb g^−1^, respectively, and had diverse values at specific locations throughout the exterior residences (Table 1). Mean Pb concentrations for the mid-yard and children’s play area were lower (80.4 and 79.0 µg Pb g^−1^, respectively), with the foundation drip line and major entryway being approximately twice as concentrated (175.4 and 142.2 µg Pb g^−1^, respectively). Vacuum-collected house dust Pb data was correlated with mean soil Pb averaged across all soil sampling locations (*r* = 0.64) (Table 3). Soil Pb from the major entryway was found to have the strongest correlation with house dust Pb (*r* = 0.66) compared to dripline, mid-yard, or children’s play area (*r* = 0.63, 0.55 and 0.56 respectively).

**Table 3:** Pearson correlation coefficients (*r*) of Pb in vacuum-collected house dust with Pb in residential soil or paint surfaces.

[Pb] correlation of dust and soil	**All soil locations**		**Specific soil collection locations**						
	Mean	Max	Major entry		Foundation dripline		Play area	Mid-yard	
	0.64	0.63	0.66		0.63		0.56	0.55	
[Pb] correlation of dust and paint	**All paint locations**			**Exterior paint**			**Interior paint**		
	Mean	Median	95th	Mean	Median	95th	Mean	Median	95th
	0.55	0.18	0.57	0.45	0.29	0.46	0.49	0.16	0.52

Relationships with residential soils are evaluated by soil sampling locations, whereas painted surfaces are separated by exterior vs. interior.

Correlations between dust Pb concentration and Pb paint levels, measured by handheld XRF as paint Pb loading (mg Pb cm^−2^), were also explored (Tables 1 and 3) [28]. House dust Pb was more strongly correlated with Pb levels in interior Pb paint (*r* = 0.49, 0.16, and 0.52 for mean, median and 95th quantile interior paint loading, respectively) than exterior paint (*r* = 0.45, 0.29 and 0.46 for mean, median and 95th quantile exterior paint loading respectively).

## Discussion

Results from the AHHS II survey indicate that homes with elevated house dust Pb concentration conditions remain present, although average house dust Pb concentrations in the United States have decreased (Table 1). Previous survey-level investigations of house dust Pb in the US, especially by dust mass, are limited. A previous HUD survey performed ~30 years ago (collection in 1993 and publication in 1998) details both dust Pb concentration and dust Pb loading data sets for 301 house dust samples [41]. The previous survey reported a mean Pb concentration of 364.2 µg Pb g^−1^ which is three times the mean concentration found in our investigation [28], suggesting a reduction in house dust Pb in the average US home between these studies. Overall decreases in house dust Pb concentrations are reasonable given efforts to reduce Pb sources over the past several decades [42]. However, homes where Pb dust concentrations remain elevated may be due to continued persistence of Pb hazards as shown in the AHHS II HUD Lead Findings report (paint, soil, etc.) [28, 42].

Another US investigation of 204 homes where children resided conducted in Rochester, New York in 1995 found substantially higher dust Pb concentrations and dust Pb loading (867.32 µg Pb g^−1^ and 130.0 µg Pb ft^−2^, respectively) than observed in our investigation [17, 26]. In addition to changes in dust Pb concentrations with time, elevated concentrations reported in the Rochester investigation may be a result of homes being in urban areas near anthropogenic sources of Pb contamination where soil and paint Pb are major contributors to house dust Pb. Outside of the US, a more recent national survey conducted by Health Canada provides a comprehensive evaluation of house dust Pb by mass and surface area (2013) [21]. Mean house dust Pb concentration for the Health Canada study was higher (210 µg Pb g^−1^); however, the Health Canada investigation specifically targeted urban areas, with approximately half of samples collected from homes within 2 km of industrial sites. Additionally, collection of the Health Canada samples was ~15 years after the aforementioned US studies, potentially representing temporal influences on house dust Pb concentration.

Across all of the aforementioned studies utilizing vacuum-collected house dust, it is important to consider that inter-study vacuum collection methodologies varied, which may limit comparisons. Such differences in collection were not assessed as part of this study. In summary, decreasing Pb concentration in house dust over time and proximity to Pb-associated anthropogenic activities (e.g., smelters, legacy contaminated urban areas, etc.) likely contributed to the lower mean dust Pb concentration observed in AHHS II.

Influence of house age and other residential characteristics on house dust Pb concentrations were further evaluated using AHHS II house age and region data. Consistent with other studies of Pb in house dust [20–22, 43], house age was found to be a significant predictor of dust Pb concentrations (Fig. 1). Notably, we observed an approximate 4-fold decrease in mean dust Pb concentration in homes built after the US CPSC issued (1978) stricter limits on house paint Pb concentrations (Fig. 1). This association is likely connected to the increased likelihood of lead-based hazards from interior and/or exterior residential sources in older homes, and/or increased time for paint to deteriorate and concentrate in house dust. The AHHS II and previous national surveys have demonstrated that paint-Pb levels increase with the age of housing, with the highest levels measured in pre-1940 housing [44, 45]. Even after ceasing use of Pb-based products, Pb is highly stable in soils and may be readily available for physical transport into homes [14, 18, 19, 46]. Additionally, home interiors once containing Pb-based paint are challenging to eliminate as contributors to house dust. Limited mass of Pb-based paint is required to incorporate into house dust and contribute to elevated dust Pb concentrations that adversely impact BLLs [17, 18, 24, 42]. This issue may be exacerbated by renovation efforts that generate fine particulate Pb-based paint deposition throughout homes, especially if sanding is performed or other proper precautions are not taken [12]. Older homes are associated with increased dust mass, which has been reported as a significant factor affecting increases in house dust Pb [21], providing another example of the connection between house age and house dust Pb.

Evaluation of census region and population density influence on house dust Pb concentrations indicate that house age is the primary factor predictive of dust Pb levels in US homes among the factors investigated as part of this study. Additional factors that may be associated with US census region or MSA beyond house age, including but not limited to population or industry density [12, 47, 48], likely have some influence on dust Pb levels in US homes (see SI), but such factors appear to be secondary to house age when evaluated at the scale of a nationwide US survey.

Although AHHS II vacuum-collected house dust is the primary focus of this study, paired dust wipe data collected by HUD was also evaluated [28]. Dust wipe data (Pb loading-basis) enabled vacuum dust Pb (concentration-basis) to be statistically related to current Pb hazard standards. While vacuum-collected house dust data represent an aggregate of all surfaces vacuumed, house dust wipe samples were collected from specific locations and surfaces in each home (Table 1) and are further described in the SI and a recent HUD report [28]. Statistically-significant correlations between vacuum-collected house dust and paired dust wipe data showcase relationships between the two dust Pb assessment methods. Correlations were stronger when wipe data was averaged by surface across rooms, which is reasonable given vacuum-collected samples in this study were a composite of dust collected throughout the home. Stronger correlations of vacuum dust Pb values with floor loading data than windowsill loading data are consistent with previous findings [17, 27, 41]. Collection of paired vacuum and dust wipe samples for dust Pb analysis from the same home is uncommon, with most investigations choosing one method to proceed with house dust Pb assessment. Lanphear et al. and Rasmussen et al. utilized both datasets to provide thorough insight into house dust Pb contamination and have used dust Pb concentration to predict dust Pb loading values, but this requires accurate determination of dust mass per residential surface area [17, 21, 27]. Given that compliance with current U.S. Pb dust hazard standards are assessed using dust wipe loading data (10 µg Pb ft^−2^ and 100 µg Pb ft^−2^ for floors and windowsills, respectively), statistically correlating vacuum-collected dust Pb concentrations to paired wipe data enabled concentration data to be related to hazard standards [28]. It is important to note, however, that while correlations were statistically significant, *r* values observed suggest the strength of this relationship is moderate in homes assessed.

Concentration data from vacuum collected dust was also related to paired loading data collected from the same home in the context of evaluating probabilities of dust Pb hazard exceedance (Fig. 3 and Tables 1 and S2). Collectively, probability of hazard exceedance and binning results suggest that homes with dust Pb wipe exceedances are likely to encounter higher Pb concentrations in house dust that exceed 400 µg Pb g^−1^. However, house dust Pb concentrations less than 400 µg Pb g^−1^ may still be of concern. Results indicate a probability of exceedance of at least ~25% at 150 µg Pb g^−1^ across both floor and windowsills combined (Fig. 3). Therefore, maximum acceptable probability of exceedance is important to consider when evaluating house dust Pb concentrations in the context of health standards.

Soil Pb has long been expected to be a mediator of house dust Pb, a relationship supported by correlations between mean soil Pb and Pb in vacuum dust (*r* = 0.64) observed in AHHS II [12, 13, 19, 20, 49, 50]. This finding provides needed data to support the long-standing assertion that soil Pb is an important mediator of house dust Pb [18, 22, 46, 50, 51]. In areas not located near industry, soil has been previously suggested as a less significant contributor to house dust, with Pb in dust expected to derive primarily from interior sources [13, 31]. Correlations between Pb in soil and dust observed in AHHS II, which includes homes across a range of population and urban densities, suggests soil is consistently important to house dust Pb. However, this does not signify that paint Pb sources are less important, rather, paint Pb and soil Pb concentration are related (Pearson correlation coefficient = 0.60; SI Table S3). The role of interconnected contributions of paint Pb and soil Pb to house dust are likely driven by house age, as decreasing house dust Pb concentration with decreasing house age was observed (Fig. 1). Soil Pb from the major entryway was found to have the strongest correlation with vacuum-collected house dust Pb (*r* = 0.66) compared to dripline, mid-yard, or children’s play area (*r* = 0.63, 0.55 and 0.56 respectively). Similar results were found for a house dust investigation in Philadelphia where elevated BLLs (µg dl^−1^) in children were found to be strongly associated soil Pb in the major entryway [22, 23]. Increased soil Pb concentration at the foundation drip line was also observed in AHHS II and was expected as this area typically has elevated Pb concentrations likely due to deterioration of exterior Pb-based paint [39, 52]. The relatively high mean Pb concentration of major entryway soil supports tracked-in sources as a potentially important pathway for soil Pb entry into the home and subsequent incorporation into dust [53]. Further investigation characterizing Pb sources and phases around the major entry area of households may provide the best prediction of house dust Pb concentration compared to other residential soil sampling locations. Additionally, targeted remediation of the entryway portions of a residence may have a substantial impact on house dust Pb, the primary contributor to elevated BLLs in children [17, 23, 24, 27].

Determination of Pb paint levels revealed that both interior and exterior paints sources are correlated with house dust Pb concentration; however, interior paint Pb had a stronger correlation (Tables 1 and 3). This observation is logical as interior Pb paint can directly contaminate indoor dust, whereas exterior Pb paint must first be tracked into the home. Stronger correlations with mean and 95th quantile values for Pb paint loading than median values suggests that associations between the Pb in paint and accumulation of Pb paint in dust may not be consistent across the range of Pb concentrations observed in each home. For example, not only may older paint in a home be more likely to have elevated Pb levels, but it may also be more likely to deteriorate, requiring only minute amounts to contribute substantially to Pb deposition in dust [17, 42].

Although vacuum- collected dust Pb was generally found to be more strongly correlated with soil Pb than paint Pb, both Pb from soil and paint are important mediators of dust Pb concentrations. Furthermore, Pb in soil and paint were strongly correlated. Specifically, mean and 95th quantile soil and paint Pb Pearson correlation coefficients were 0.60 and 0.69, respectively (SI Table S3), suggesting paint Pb contributes to soil Pb as exterior paint deteriorates and deposits into soil systems [52] before infiltrating homes and accumulating in house dust. Other major contributors of soil Pb, in addition to paint Pb, include leaded gasoline, Pb smelters, and other industrial activities [52]. The strong correlation of soil and paint Pb provides valuable insight showcasing interconnection between Pb sources that may concentrate in house dust. Pb phases indicative of Pb-based paint are still found in prior Pb speciation investigations of both soils and house dust and likely contribute to sorbed Pb concentrations in soils as paint containing Pb phases degrade [14, 30, 31]. Additionally, identification of paint Pb species in house dust may not necessarily be an indicator of contamination originating from within the home, as exterior paint Pb surfaces are, on average, more concentrated than interior sources (Table 1) and are correlated with soil Pb (SI Table S3). This finding is critical for future speciation analyses probing Pb sources within and around residences, where limited investigations have revealed the presence of Pb phases commonly attributed to white paint pigment [14, 30].

House dust Pb remains a persistent issue contributing to increased BLLs in the United States and globally [5]. The AHHS II study provides a window into the current state of Pb in and around residences. Here we evaluated the relationship between house dust Pb concentrations and two common residential Pb sources: soil and Pb-based paint. Despite the banning of Pb-based paint ~45 years ago, results of this survey suggest that dust Pb concentrations in homes continue to be influenced by Pb-based paint and soil contaminated with Pb (SI Table S3), in particular through soil transport from the major entryway. House dust Pb concentrations and loading have decreased with time, but Pb sequestered in soils, in conjunction with Pb-based paint, continue to be mediators of house dust Pb contamination. This is especially true for older homes built prior to the banning of Pb-based paint (pre-1978) in residential and public properties, where legacy Pb-based paint in residence interior and exteriors is more likely. Pb readily sorbs to many environmental constituents that facilitate stability in soil systems [14, 54, 55] and may be available for physical transport into home interior; however, this stability is not expected to persist upon ingestions and/or inhalation [14, 56, 57]. Sorbed Pb and paint Pb phases (e.g., cerussite, hydrocerussite) have been found to be highly bioavailable [14]. Therefore, results presented here suggest that soil Pb remediation, potentially via in situ chemical remediation methods [54, 58], may be an important path to mitigating further Pb contamination of house dust via the soil to dust pathway.

Current USEPA hazard standards of house dust Pb are based on Pb loading data (µg Pb ft^−2^) using house dust wipes; however, our results demonstrate that probability of dust Pb loading exceeding hazard standards can be statistically estimated using house dust Pb concentration data from the same home. We observed an ~25% probability of exceedance for dust Pb concentrations of 150 µg Pb g^−1^. Therefore, the predictive model (Fig. 3) may be useful in cases where only house dust Pb concentration data is available and improved with future paired dust Pb concentration and loading datasets. Future investigations probing specific Pb phases in house dust and relationships to residential sources will be essential to assessing exposure and developing effective remediation strategies.

Dust samples from household vacuum bags can provide a cost-effective measure of contaminant concentrations [26, 59]. While important, there are weaknesses to using vacuum-collected house dust. An important disadvantage of household vacuum samples is the lack of information on dust age and provenance (i.e., area sampled) required for calculating dust and elemental loading rates. Vacuum collection methods are generally more variable than wipe methods, and don’t allow for dust Pb to be evaluated per surface area. This limits the direct usage of vacuum-collected house dust to current hazard standards based on Pb loading values.

AHHS II included collection of additional data shown in previous studies to relate to dust Pb levels in homes, including socio-economic data. Such data was collected by means of a questionnaire completed by the homeowner or representative, with responses to questions being completely voluntary. Upon review of the available data, we determined there was insufficient socio-economic data available to include these as factors assessed in this study. This also limited the number of variables that could be considered in multivariate models relating factors to vacuum-collected dust Pb concentrations.

Collection of dust from the homeowner’s vacuum provided mass quantities necessary for inorganic analyses and advanced characterization techniques that are either more challenging or unachievable using wipe methods. Using dust from homeowner’s vacuum also provides a quick, easy, low-cost method and low burden effort on the homeowner for collecting dust from homes. Therefore, results of Pb in vacuum-collected house dust presented here may facilitate future Pb dust investigations, particularly in larger surveys where such benefits may be of particular value.

### Supplementary information

Supporting Information

## Data Availability

Data presented in this publication are available from the corresponding author on reasonable request.
