# Supplementary material for: United States house dust Pb concentrations are influenced by soil, paint, and house age: insights from a national survey
Source: J Expo Sci Environ Epidemiol. 2024 Mar 28;34(4):709–17. doi: 10.1038/s41370-024-00655-0 (PMC11303246; doi:10.1038/s41370-024-00655-0)
Supplement: Supplementary file 1 — Supporting Information [file 41370_2024_655_MOESM1_ESM.docx]

**Supporting Information**

**United States house dust Pb concentrations are influenced by soil, paint, and house age: Insights from a national survey**

Tyler D. Sowers^1*^, Clay M. Nelson^2^, Matthew D. Blackmon^1^, Kevin Li^3^, Marissa L. Jerden^4^, Alicia M. Kirby^5^, Kasey Kovalcik^1^, David Cox^6^, Gary Dewalt^6^, Warren Friedman ^7^, Eugene A. Pinzer^7^, Peter J. Ashley^7^, Karen D. Bradham^1^

^1^Center of Environmental Measurement and Modeling, Office of Research and Development, US Environmental Protection Agency, Research Triangle Park, North Carolina 27711, US.

^2^BioGeoChem Scientific, Austin, Texas 78748, US.

^3^Independent Researcher, Lansing, MI 48915.

^4^ Jacobs Technology, Inc., 109 T.W. Alexander Drive, RTP, North Carolina 27711, US.

^5^Oak Ridge Associated Universities, Oak Ridge, Tennessee 37830, US.

^6^QuanTech, 6110 Executive Blvd Suite 206, Rockville MD 20852

^7^Office of Lead Hazard Control and Healthy Homes, Department of Housing and Urban Development, Washington, District of Columbia 20410, US.

Number of Pages: 8

Number of Tables: 3

Number of Figures: 2

*Corresponding author - Office of Research and Development, United States Environmental Protection Agency, Research Triangle Park, North Carolina 27711, US, [sowers.tyler@epa.gov](mailto:sowers.tyler@epa.gov)

**American Healthy Homes II Background**

Prior to sample collection, household members were instructed to refrain from cleaning the floors of their homes and emptying their vacuum for a month.

**Digestion Methods**

Of the 346 house dust samples evaluated, 169 were performed in-house while 177 were sent to a third-party contractor for digestion following the same procedure and QC criteria. Due in part to unanticipated analytical throughput challenges resulting from the COVID-19 pandemic, both hotblock and microwave digestion methods were used to measure dust Pb concentrations, and a portion of dust samples were analyzed using a contracted lab. A comparison study was performed to ensure both labs and methods resulted in corresponding Pb data (SI Table S1).

**Dust Wipe Methods**

Two wipe samples were collected from in each of the rooms, one from a random windowsill and one from the center of the largest open area on the floor. Also included were collection of one field blank and one sample from the floor in the center of the doorway to the major entrance to the housing unit. One square foot templates were used for floor samples. The entire interior sill area was wiped for windowsill samples. The surface type wiped for floor and window samples, carpet pile depth (for carpeted surfaces), window treatments, surface area wiped, and proximity of floor samples to doors, windows, and traffic patterns were recorded. Further detail on the aforementioned methods may be found in the AHHS II HUD report.^1^

**XRF Methods**

The AHHS II Risk Assessor was responsible for conducting X-Ray Fluorescence (XRF) with a portable XRF. Testing of interior and exterior paint to determine lead levels was completed using the Viken Pb200i XRF instrument, which recorded all lead readings electronically and was programmed to also record the component type tested for each reading. The XRF was programmed so that a “percent deteriorated paint” for the component was required to be entered into the instrument before each reading was taken. The possible entries were: 0% (no deteriorated paint); 1-10%; 11-25%; 25-50%; 51-75%; 76-90%; 91-99%; and, 100% (all paint on the component was deteriorated). Further detail on the aforementioned methods may be found in the AHHS II HUD report.^1^

**Dust Loading Data (i.e., Dust Wipe Data)**

For paired wipe sample data, mean house dust Pb per ft^2^ was 22.3 µg Pb ft^-2^ across all surfaces. Mean dust wipe data collected from floors was 5.2 µg Pb ft^-2^, well below the overall mean across all sampling locations. Conversely, windowsills were found to have a mean loading value of 51.0 µg Pb ft^-2^, an order of magnitude greater than both floors and the overall dataset. For the 1998 HUD study, mean dust Pb loading of 19.3 µg Pb ft^-2^ was reported. Differences in mean house dust µg Pb ft^-2^ between this study and the 1998 HUD study were not statistically significant. Multiple factors may contribute to a lack of decrease in mean dust Pb per ft^2^ between the two studies, including increased dust mass, location of sampling, and contribution of soil and/or paint Pb.^2-5^ Despite significant decline in the presence of Pb-based paint in homes, relatively minute amounts that deteriorate may have a significant impact on dust Pb deposition.^6^ Hard-surface floorings were evaluated in the 1998 study whereas windowsills were included in the present AHHS II survey. The 1998 HUD study and the Rochester investigation found windowsills to have the greatest quantity of dust Pb loading which is congruent with AHHS II findings.^7, 8^

**Residential Indicators of House Dust Pb**

Using the Northeast region as a baseline for comparison, the multiple regression model found that the South and West regions were significantly lower by 33.4% (95% CI: -9.1 – -51%, p=0.01) and 33.9% (95% CI: -7.4 – -53%, p=0.02), respectively, suggesting there may be factors beyond house age that influence regional differences in dust Pb concentrations (e.g., traffic density and legacy impacts of leaded-gasoline).^9, 10^ The effect of these differences was small compared to the power of our analysis and dataset, however, as additional pairwise comparisons by the Tukey post-hoc test between the model-estimated census region means (holding house age at the study-wide mean) suggest these differences are marginally significant (p<0.10).

**Table S1.** Lab and method comparison for house dust Pb analyses. Results showcases congruence between EPA and a contracted lab (RTI), as well as between microwave and hotblock methods. Mean repeatability was calculated by dividing both means and reporting the resulting recovery as a percentage.

|  | **Inter-lab** | **Intra-lab** |
| --- | --- | --- |
|  | **SRM 2710a Hotblock (EPA:RTI)** | **Microwave:Hotblock (EPA)** |
| Mean Repeatability | 94.36% | 107.98% |
| N-value | 10 | 6 |

**Table S2.** Pearson correlation coefficients for vacuum-collected house dust Pb data compared to either all or specific residential locations of dust wipe Pb data collection.

| **Vacuum & Wipe data** | **all floor** | | | **all window** | | | **kitchen** | | **common living area** | | **bedroom** | | **other** | | **entryway** |
| --- | --- | --- | --- | --- | --- | --- | --- | --- | --- | --- | --- | --- | --- | --- | --- |
|  | **mean** | **median** | **95th QT*** | **mean** | **median** | **95th QT*** | **floor** | **window** | **floor** | **window** | **floor** | **window** | **floor** | **window** | **floor** |
| **Pearson correlation coefficient** | 0.48 | 0.41 | 0.49 | 0.41 | 0.4 | 0.41 | 0.31 | 0.25 | 0.39 | 0.37 | 0.4 | 0.39 | 0.43 | 0.35 | 0.44 |

*QT=Quantile

**Table S3.** Pearson correlation coefficient of soil mean or max with either all painted surfaces or solely exterior.

| **soil mean and…** | | | | **soil max and…** | | | |
| --- | --- | --- | --- | --- | --- | --- | --- |
| **all paint** | | **exterior only** | | **all paint** | | **exterior only** | |
| **mean** | **95th QT*** | **mean** | **95th QT*** | **mean** | **95th QT*** | **mean** | **95th QT*** |
| 0.6 | 0.69 | 0.58 | 0.59 | 0.6 | 0.68 | 0.58 | 0.6 |

*QT=Quantile


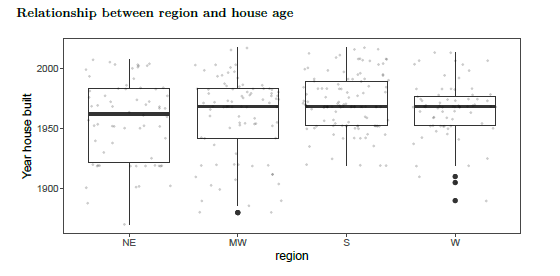


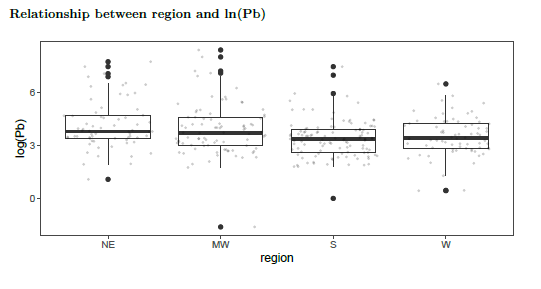


**Figure S1.** AHHS II data showcasing year houses were built (top) or log([Pb]) of house dust (bottom) across the Northeast (NE), Midwest (MW), South (S), and West (W) United States census regions.

| 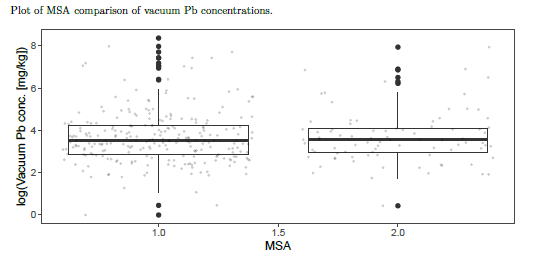 |
| --- |
| **MSA non-MSA** |

**Figure S2.** Plot of log([Pb]) for house dust collected from homes located in metropolitan statistical areas (MSAs) versus non-MSA homes.

**References**

1. HUD. American Healthy Homes Survey II Lead Findings. In. U.S. Department of Housing and Urban Development ed: Office of Lead Hazard Control and Healthy Homes, 2021. https://www.hud.gov/sites/dfiles/HH/documents/AHHS%20II_Lead_Findings_Report_Final_29oct21.pdf

2. Bevington C, Gardner HD, Cohen J, Henning C, Rasmussen PE Relationship between residential dust-lead loading and dust-lead concentration across multiple North American datasets. Building and Environment 2021; 188: 107359.

3. Lanphear BP, Emond M, Jacobs DE, Weitzman M, Tanner M, Winter NL *et al* A side-by-side comparison of dust collection methods for sampling lead-contaminated house dust. Environmental research 1995; 68: 114-123.

4. Rasmussen PE, Levesque C, Chénier M, Gardner HD, Jones-Otazo H, Petrovic S Canadian House Dust Study: Population-based concentrations, loads and loading rates of arsenic, cadmium, chromium, copper, nickel, lead, and zinc inside urban homes. Science of the total environment 2013; 443: 520-529.

5. Sutton PM, Athanasoulis M, Flessel P, Guirguis G, Haan M, Schlag R *et al* Lead levels in the household environment of children in 3 high-risk communities in California. Environmental research 1995; 68: 45-57.

6. Jacobs DE, Clickner RP, Zhou JY, Viet SM, Marker DA, Rogers JW *et al* The prevalence of lead-based paint hazards in US housing. Environmental health perspectives 2002; 110: A599-A606.

7. Lanphear BP, Weitzman M, Winter NL, Eberly S, Yakir B, Tanner M *et al* Lead-contaminated house dust and urban children's blood lead levels. American Journal of Public Health 1996; 86: 1416-1421.

8. USEPA. *Risk Analysis to Support Standards for Lead in Paint, Dust, and Soil. Appendices B to G: Health Effects Associated with Exposure to Lead and Internal Lead Doses in Humans*. US Environmental Protection Agency: Washington, DC, 1998.

9. Mielke HW, Gonzales CR, Smith MK, Mielke PW The urban environment and children's health: soils as an integrator of lead, zinc, and cadmium in New Orleans, Louisiana, USA. Environmental research 1999; 81: 117-129.

10. Mielke HW, Reagan PL Soil is an important pathway of human lead exposure. Environmental health perspectives 1998; 106: 217-229.
